# Supplementary material for: nCov2019: an R package for studying the COVID-19 coronavirus pandemic
Source: PeerJ. 2021 Jun 10;9:e11421. doi: 10.7717/peerj.11421 (PMC8199916; doi:10.7717/peerj.11421)
Supplement: Supplemental Information 1 [file peerj-09-11421-s001.pdf]

nCov2019: A R package for studying COVID-19 coronavirus outbreak

Tianzhi Wu, Erqiang Hu, Patrick Tung, Xijin Ge, Guangchuang Yu

2021-03-25

- Installation
- Statistic query
  - Global data
  - Latest data
  - Historical data
  - Vaccine and therapeutics data
- Visualization
- Animations plot
- Other plots
- Heatmap for cases per country
- Dashboard
- statistic item explanation
- Session Info

To provide convenient access to epidemiological data on the coronavirus outbreak, we developed an R package, nCov2019 (https://github.com/yulab-smu/nCov2019 (https://github.com/yulab-smu/nCov2019)). Besides detailed basis statistics, it also includes information about vaccine development and therapeutics candidates. We redesigned the function plot() for geographic maps visualization and provided a interactive shiny app. These analytics tools could be useful in informing the public and studying how this and similar viruses spread in populous countries.

Our R package is designed for both command line and dashboard interaction analysis, As show in diagram, while `dashboard()` is the main entry for the GUI explore part, the `query()` is the main function used in CLI explore part, 5 types of data were contain in its return result. data type were explain in **Statistic query** part.

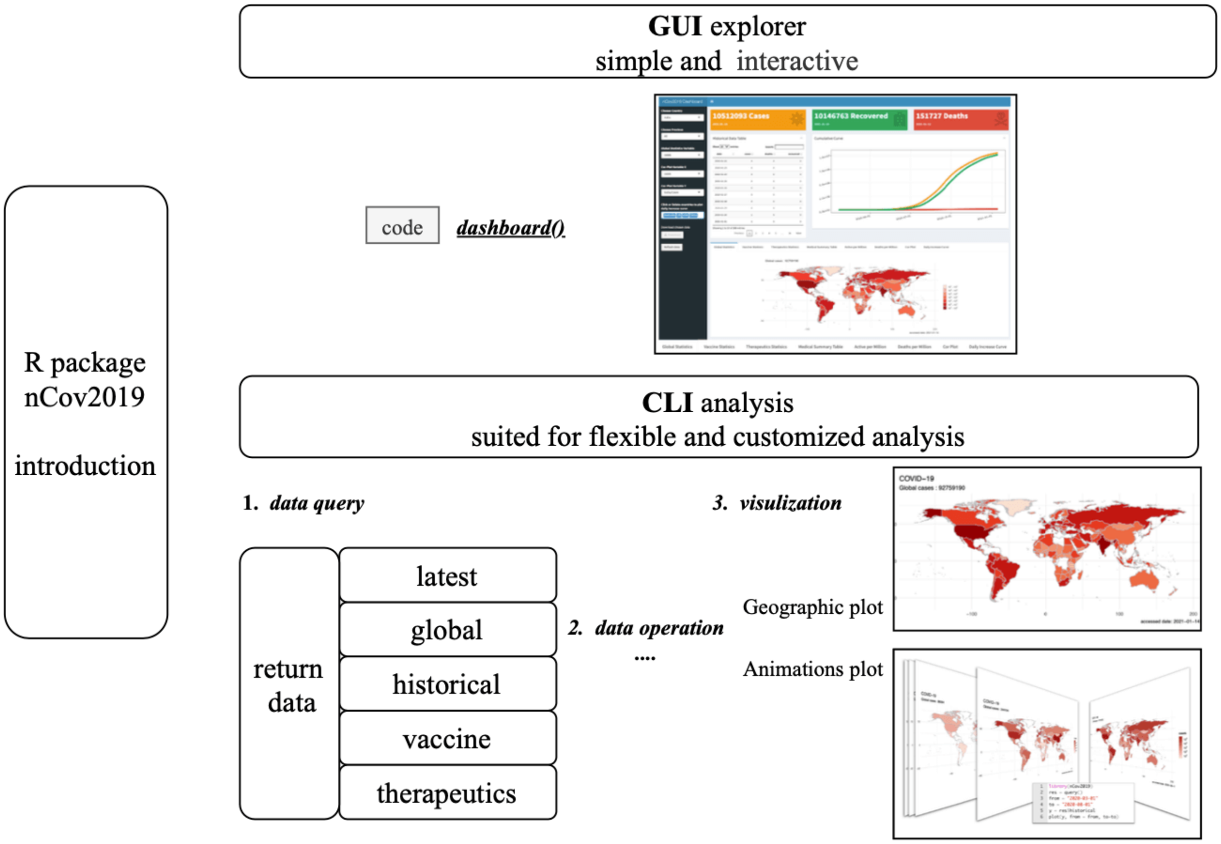

introduction

Installation

To start off, users could utilize the ‘remotes’ package to install it directly from GitHub by running the following in R:

```
remotes::install_github("yulab-smu/nCov2019", dependencies = TRUE)
```

Statistic query

Data query is simple as one command:

```
library("nCov2019")
res <- query()
```

```
## Querying the latest data...
```

```
## last update: 2021-03-25
```

```
## Querying the global data...
```

```
## Gloabl total  125537800  cases; and  2758723  deaths
## Gloabl total affect country or areas: 221
## Gloabl total recovered cases: 71438
## last update: 2021-03-25
```

```
## Querying the historical data...
```

```
## Querying the vaccine data...
```

```
## Total Candidates Programs : 51
```

```
## Querying the therapeutics data...
```

```
## Total Candidates Programs : 54
```

```
## Query finish, each time you can launch query() to reflash the data
```

This may take seconds to few minutes, which depend on the users' network connection, if the user connection is broken, a local stored version data will be used for demo.

The result returned by `query()` function will contains 5 types of statistic:

```
names(res)
```

```
## [1] "latest"       "global"       "historical"   "vaccine"      "therape
utics"
```

- `global` The global overall summary statistic
- `latest` The global latest statistic for all countries
- `historical` The historical statistic for all countries
- `vaccine` The current vaccine development progress
- `therapeutics` The current therapeutics development progress

The `query()` only need to be performed once in a session, print each of statistic objects, users could get their update time. And for the `vaccine` and `therapeutics` query results, print them will return the candidates number.

Global data

The query result of global status will contain a data frame with 21 types of statistic, which have detail explanation on the bottom of this documents. And `summary(x)` will return overview of global status.

```
x = res$global
x$affectedCountries # total affected countries
```

```
## [1] 221
```

```
summary(x)
```

```
## Gloabl total  125537800  cases; and  2758723  deaths
## Gloabl total affect country or areas: 221
## Gloabl total recovered cases: 71438
## last update: 2021-03-25
```

Latest data

Here is the example for operating latest data. once again, all data have queried and store in `res`.

```
x = res$latest
```

And then `print(x)` will return the update time for the latest data

```
print(x) # check update time
```

```
## last update: 2021-03-25
```

To subset latest data could be easily done by using `[`. `x["Global"]` or x `["global"]` will return the data frame for all countries but users could determine a specific country, such as:

```
head(x["Global"]) # return all global countries.
```

	country <chr>	cases <int>	deat... <int>	recovered <int>	active <int>	todayCases <int>	todayDeaths <int>	todayRe
1	Brazil	12227179	301087	10689646	1236446	90564	2244	
2	USA	30704292	558422	23132879	7012991	66538	1405	
3	India	11787013	160726	11229591	396696	53419	249	
4	France	4378446	93180	283507	4001759	33389	272	
5	Poland	2120670	50340	1707846	362484	29977	575	
6	Turkey	3091282	30462	2881643	179177	29762	146	

6 rows | 1-10 of 12 columns

```
x[c("USA","India")] # return only for USA and India
```

	country<br><chr>	cases<br><int>	deat...<br><int>	recovered<br><int>	active<br><int>	todayCases<br><int>	todayDeaths<br><int>	todayRe
2	USA	30704292	558422	23132879	7012991	66538	1405	
3	India	11787013	160726	11229591	396696	53419	249	

2 rows | 1-10 of 12 columns

The data is order by "todayCases" column, users could sort them by other order.

```
df = x["Global"]
head(df[order(df$cases, decreasing = T),])
```

	country<br><chr>	cases<br><int>	deat...<br><int>	recovered<br><int>	active<br><int>	todayCases<br><int>	todayDeaths<br><int>	todayR
2	USA	30704292	558422	23132879	7012991	66538	1405	
1	Brazil	12227179	301087	10689646	1236446	90564	2244	
3	India	11787013	160726	11229591	396696	53419	249	
13	Russia	4483471	96219	4098400	288852	8861	401	
4	France	4378446	93180	283507	4001759	33389	272	
24	UK	4312908	126382	3729155	457371	5605	98	

6 rows | 1-10 of 12 columns

As for the latest data, it provides 11 types of main information by default, but 12 more statistic type are provided in the "latest$detail", they also have corresponding explanation on the bottom.

```
x = res$latest
head(x$detail)  # more detail data
```

	updated<br><chr>	country<br><chr>	countryInfo<br><data.frame>	cases<br><int>	todayCases<br><int>	deat...<br><int>	toda
1	2021-03-25	Brazil	<data.frame [6 × 6]>	12227179	90564	301087	
2	2021-03-25	USA	<data.frame [6 × 6]>	30704292	66538	558422	
3	2021-03-25	India	<data.frame [6 × 6]>	11787013	53419	160726	
4	2021-03-25	France	<data.frame [6 × 6]>	4378446	33389	93180	
5	2021-03-25	Poland	<data.frame [6 × 6]>	2120670	29977	50340	
6	2021-03-25	Turkey	<data.frame [6 × 6]>	3091282	29762	30462	

6 rows | 1-9 of 24 columns

Historical data

Historical data is useful in retrospective analysis or to establish predictive models, the operation is similar as latest data, user could get the data frame for all countries or some specific countries within `c()` vector, such as `head(Z[c(country1,country2,country3)])`

```
Z = res$historical
print(Z) # update time
```

```
## last update: 2021-03-24
```

```
head(Z["Global"])
```

	country	date	cases	deaths	recovered
	<chr>	<date>	<int>	<int>	<int>
1	Afghanistan	2020-01-22	0	0	0
193	Afghanistan	2020-01-23	0	0	0
385	Afghanistan	2020-01-24	0	0	0
577	Afghanistan	2020-01-25	0	0	0
769	Afghanistan	2020-01-26	0	0	0
961	Afghanistan	2020-01-27	0	0	0

6 rows

```
head(Z[c("China","UK","USA")])
```

	country	date	cases	deaths	recovered
	<chr>	<date>	<int>	<int>	<int>
37	China	2020-01-22	548	17	28
229	China	2020-01-23	643	18	30
421	China	2020-01-24	920	26	36
613	China	2020-01-25	1406	42	39
805	China	2020-01-26	2075	56	49
997	China	2020-01-27	2877	82	58

6 rows

For the following countries, we provide detail province data, which can be obtained in a similar way but within `[` operation: `head(Z[country,province])`

- `Australia` `Canada` `China` `Denmark` `France` `Netherlands`

```
head(Z['China','hubei'])
```

	country<br><chr>	province<br><chr>	date<br><date>	cases<br><int>	deaths<br><int>	recovered<br><int>
34	China	hubei	2020-01-22	444	17	28
119	China	hubei	2020-01-23	444	17	28
204	China	hubei	2020-01-24	549	24	31
289	China	hubei	2020-01-25	761	40	32
374	China	hubei	2020-01-26	1058	52	42
459	China	hubei	2020-01-27	1423	76	45

6 rows

For users' own historical data, we provide a `convert()` function, users could convert other data into class of nCov2019History data, and then explore in nCov2019:

```
userowndata <- read.csv("path_to_user_data.csv")
# userowndata, it should contain these 6 column:
# "country","province","date","cases","deaths","recovered"
Z = convert(data=userowndata)
head(Z["Global"])
```

Vaccine and therapeutics data

Users could check for the vaccine or therapeutics developing status. Let x be the vaccine or therapeutics query result, then `summary()` will return the summary of their trial phase, and x["all"] or x["All"] will return the summary information, such as mechanism, trial Phase, institutions and so on. Then the detail background info will return with provided id, for example x[ID="id3"] or simple as x["id3"]. The same operation could apply to therapeutics data.

```
X <- res$vaccine
summary(X)
```

	phase<br><chr>	candidates<br><chr>
1	Phase 3	10
2	Phase 2/3	3
3	Phase 2	2
4	Phase 1/2	9
5	Phase 1	13

	phase	candidates
	<chr>	<chr>
6	Pre-clinical	14

6 rows

```
head(X["all"])
```

	id	candidate	mechanism
	<chr>	<chr>	<chr>
1	id1	BNT162	mRNA-based vaccine
2	id2	mRNA-1273	mRNA-based vaccine
3	id3	Ad5-nCoV	Recombinant vaccine (adenovirus type 5 vector)
4	id4	AZD1222	Replication-deficient viral vector vaccine (adenovirus from chimpanzees)
5	id5	CoronaVac	Inactivated vaccine (formalin with alum adjuvant)
6	id6	Covaxin	Inactivated vaccine

6 rows | 1-4 of 7 columns

```
# check for the details about the mRNA-based vaccine, id3
X[ID="id3"]
```

```
## [1] "Background: China&rsquo;s CanSino Biologics has developed a recombi
nant novel coronavirus vaccine that incorporates the adenovirus type 5 vect
or (Ad5) named Ad5-nCoV. Trials: Multiple trials are in various stages of r
ecruitment and completion: - A Phase 1 clinical trial in China of 108 parti
cipants between 18 and 60 years old who will receive low, medium, and high
doses of Ad5-nCoV is active, but not recruiting (NCT04313127). - A Phase 1
trial in China is evaluating intramuscular vaccination and mucosal vaccinat
ion of Ad5-nCoV across two doses (NCT04552366). - A Phase 1/2 trial of up t
o 696 participants in Canada (NCT04398147). - A Phase 2 double-blind, place
bo-controlled trial of up to 508 participants in China (NCT04341389) is act
ive, but not recruiting. - A Phase 2b trial in China evaluating safety and
immunogenicity of Ad5-nCoV in participants 6 years and older (NCT04566770).
- A Phase 3 trial in Russia of up to 500 participants across multiple study
centers (NCT04540419). - A Phase 3 trial of up to 40,000 participants inter
nationally, including Pakistan, Saudi Arabia and Mexico (NCT04526990). Outc
omes: A single dose of Ad5-nCoV protected against upper respiratory infecti
on of SARS-CoV-2 in ferrets, according to a paper published 14 August in Na
ture Communications. Results from a Phase 1 trial show a humoral and immuno
genic response to the vaccine, according to a paper published in The Lance
t. Adverse reactions such as pain (54%), fever (46%), fatigue (44%), headac
he (39%), and muscle pain (17%) occurred in 83% of patients in the low and
medium dose groups and 75% of patients in the high dose group. In the Phase
2 trial, neutralizing antibodies and specific interferon γ enzyme-linked im
munospot assay responses were observed at all dose levels for most particip
ants. Status: On 25 June, China's Central Military Commission announced the
military had been approved to use Ad5-nCoV for a period of 1 year, accordin
g to reporting in Reuters."
```

```
X <- res$therapeutics
summary(X)
```

	phase<br><chr>	candidates<br><chr>
1	Phase 3	14
2	Phase 2/3	12
3	Phase 2	14
4	Phase 1/2/3	1
5	Phase 1b	4
6	Phase 2/3/4	1
7	Phase 2b/3	2
8	Phase 3/4	1
9	Phase 2/4	1
10	Phase 1/2	1

1-10 of 12 rows Previous **1** 2 Next

```
head(X["All"])
```

	id <chr>	medicationClass <chr>	tradeName <chr>	developerI <chr>
1	id1	IL-6 receptor agonist	Actemra (tocilizumab)	Roche
2	id2	Antirheumatic agent	Bucillamine	Revive The
3	id3	Monoclonal antibody	Bamlanivimab (LY-CoV555)	Lilly
4	id4	Monoclonal antibody	VIR-7831 (GSK4182136)	Vir Biotech
5	id5	Monoclonal antibody	Mavrilimumab	Kiniksa Pha
6	id6	Antibody cocktail	Casirivimab/imdevimab (REGN-COV2)	Regeneron

6 rows | 1-5 of 8 columns

```
X[ID="id1"]
```

```
## [1] "Background: Actemra is a indicated to treat autoimmune diseases suc
h as rheumatoid arthritis as well as cytokine release syndrome. Research fr
om China has shown Actemra may be an effective treatment for patients with
severe cases of COVID-19.  Trials: Actemra is being evaluated in the follow
ing high-profile trials: COVACTA (NCT04320615) and EMPACTA (NCT04372186). T
he Hôpitaux de Paris (CORIMUNO-19) is assessing Actemra in a trial for COVI
D-19 associated pneumonia (NCT04331808) in a Phase 2 trial.  Outcomes: Evid
ence is beginning to point to Actemra having a beneficial outcome for COVID
-19 patients in some, but not all, scenarios. Evidence for benefit: - Resul
ts from EMPACTA indicate Actemra reduced the need for mechanical ventilatio
n in patients with COVID-19 associated pneumonia. In EMPACTA, 12.0% of pati
ents receiving Actemra received mechanical ventilation compared with 19.3%
of patients in the placebo group (P = .04); however, Actemra did not improv
e rates of survival, according to data published in the New England Journal
of Medicine. - Preliminary results from CORIMUNO-19 showed Actemra “improve
s significantly clinical outcomes” of pneumonia associated with COVID-19. -
The drug may also improve survival in patients with cytokine release syndro
me, according to a study in CHEST. - Results from the University of Michiga
n published in the journal Clinical Infectious Diseases showed a 45% reduct
ion in hazard of death for COVID-19 patients and improved status compared w
ith patients who did not receive the drug. - In a multicenter cohort study
of 4,485 adults with COVID-19 published in JAMA Internal Medicine, research
ers found a lower risk of mortality in adults who received Actemra within 2
days of admission to the ICU compared with patients who did not receive Act
emra as part of their care. Evidence showing mixed results: - Researchers o
n behalf of the Niguarda COVID-19 Working Group released a comparative anal
ysis in the Journal of Infection that noted Actemra is potentially effectiv
e, but recommended caution when using the drug. - A randomized, double-blin
d, placebo-controlled trial published in the New England Journal of Medicin
e by researchers at Massachusetts General Hospital found Actemra was not ef
fective in reducing need for intubation, disease progression, or death but
left open the opportunity that the drug did carry some benefit due to wide
confidence intervals in comparisons of efficacy. - CORIMUNO-19: There were
a lower number of patients hospitalized with COVID-19 and moderate-to-sever
e pneumonia taking Actemra who required noninvasive ventilation, intubatio
n, or died at 14 days compared with placebo, but Actemra did not meet the p
rimary outcome of reducing clinical progression scores by 5 days after star
ting treatment. Evidence showing no benefit: - In the COVID-BioB Study, pat
ients who received Actemra instead of standard of care had improved clinica
l outcomes (69% vs. 61%; P = .61) and reduced mortality (15% vs. 33%; P = .
15), but neither result was statistically significant. - Results posted in
medRxiv by researchers at the University of North Carolina, Chapel Hill, sh
owed that of 11 patients with severe COVID-19 requiring ventilation, Actemr
a reduced C-reactive protein levels but did not result in significant impro
vement in temperature and oxygen requirements. - An Italian study sponsored
by the Italian Medicine Agency (AIFA) was stopped after Actemra failed to p
erform better than standard of care in reducing respiratory symptoms, inten
sive care visits and mortality. - Roche also provided an update for COVACTA
indicating the drug did not meet its primary or secondary endpoints of impr
oved clinical status and reduced mortality.  Status: COVACTA has been compl
eted; EMPACTA and CORIMUNO-19 are active, but not recruiting."
```

Visualization

We provide a visualization function as a redesign "plot".

```
plot(
  x,
  region = "Global",
  continuous_scale = FALSE,
  palette = "Reds",
  date = NULL,
  from = NULL,
  to = NULL,
  title = "COVID-19",
  type = "cases",
  ...
)
```

Here, type could be one of "cases","deaths","recovered","active","todayCases","todayDeaths", "todayRecovered","population" and "tests". By default, color palette is "Reds", more color palettes can be found here: palette (https://www.r-graph-gallery.com/38-rcolorbrewers-palettes.html)

To get the overview for the latest status, the mini code required is as below:

```
X <- res$latest
plot(X)
```

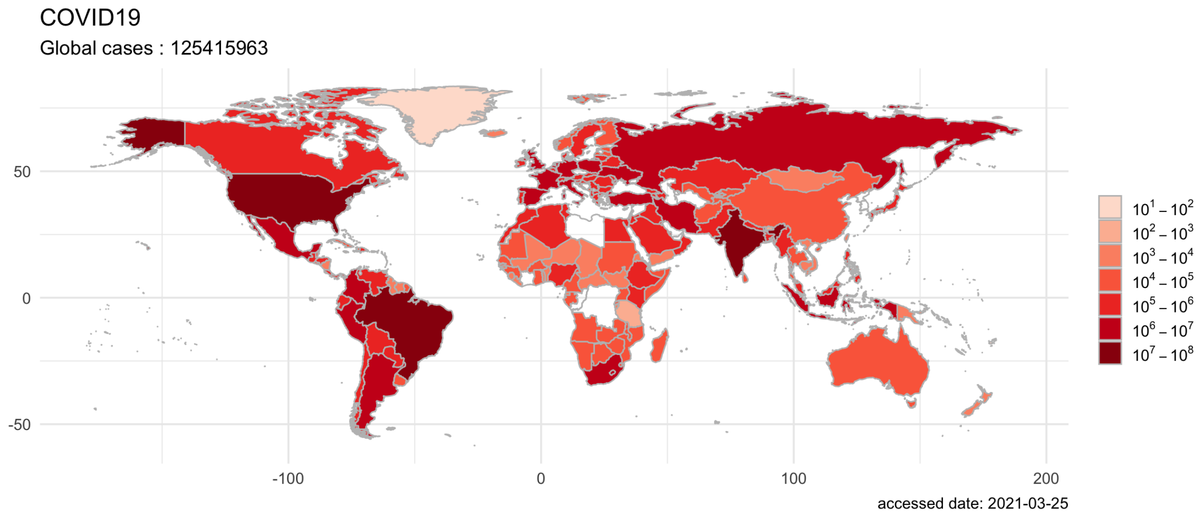

Or To get the overview for the detection testing status,

```
plot(X, type="tests",palette="Green")
```

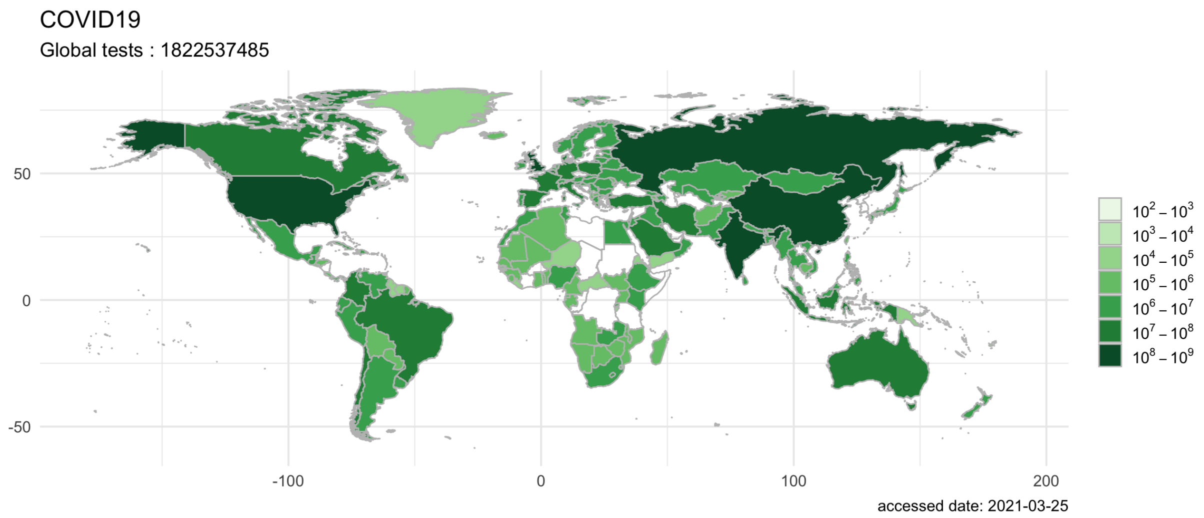

It could be also intuitively compare the number of new confirmed cases per day among different countries.

```
library(ggplot2)
library(dplyr)
```

```
##
## Attaching package: 'dplyr'
```

```
## The following objects are masked from 'package:stats':
##
##     filter, lag
```

```
## The following objects are masked from 'package:base':
##
##     intersect, setdiff, setequal, union
```

```
X <- res$historical
tmp <- X["global"] %>%
  group_by(country) %>%
  arrange(country,date) %>%
  mutate(diff = cases - lag(cases, default =  first(cases))) %>%
  filter(country %in% c("Australia", "Japan", "Italy", "Germany",  "China"
))

ggplot(tmp,aes(date, log(diff+1), color=country)) + geom_line() +
  labs(y="Log2(daily increase cases)") +
  theme(axis.text = element_text(angle = 15, hjust = 1)) +
  scale_x_date(date_labels = "%Y-%m-%d") +
  theme_minimal()
```

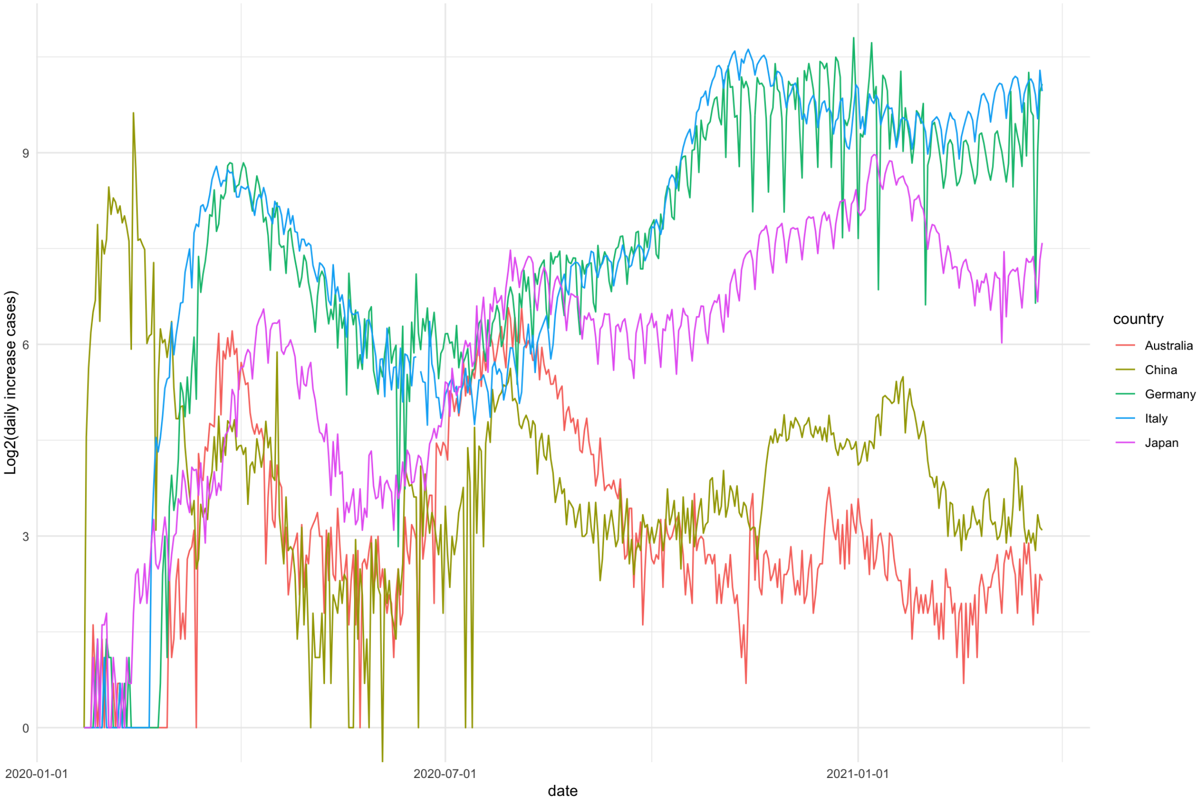

user could also plot the outbreak map on the past time with historical data by specify a date in function plot().

```
Y <- res$historical
plot(Y, region="Global" ,date = "2020-08-01", type="cases")
```

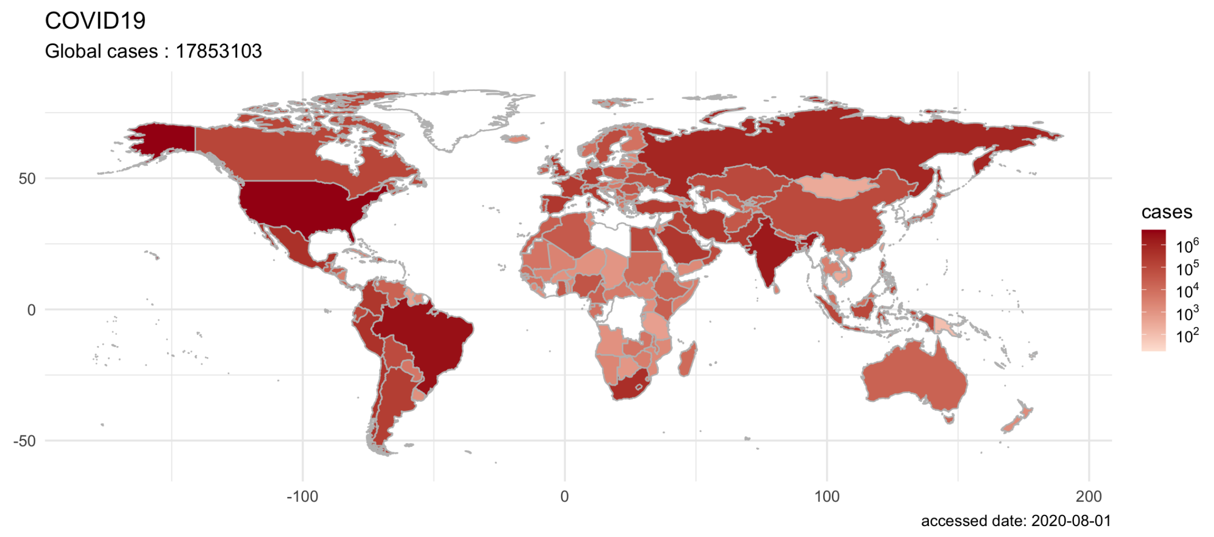

Animations plot

Animated world-wide epidemic maps could be generated in the similar way. This is the example to draw a spread animation from 2020-03-01 to 2020-08-01, with little code.

```
library(nCov2019)
res = query()
from = "2020-03-01"
to = "2020-08-01"
y = res$historical
plot(y, from = from, to=to)
```

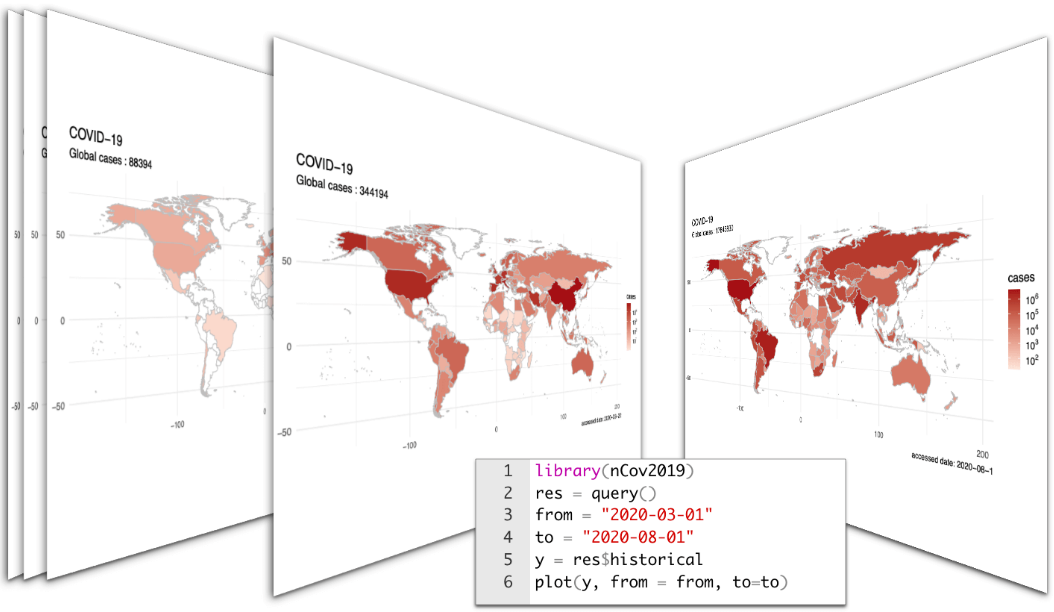

overview

Other plots

If you wanted to visualize the cumulative summary data, an example plot could be the following:

```
library(ggplot2)
x <- res$historical
d = x['Japan' ] # you can replace Anhui with any province
d = d[order(d$cases), ]

ggplot(d,
       aes(date, cases)) +
  geom_col(fill = 'firebrick') +
  theme_minimal(base_size = 14) +
  xlab(NULL) + ylab(NULL) +
  scale_x_date(date_labels = "%Y/%m/%d") +
  labs(caption = paste("accessed date:", max(d$date)))
```

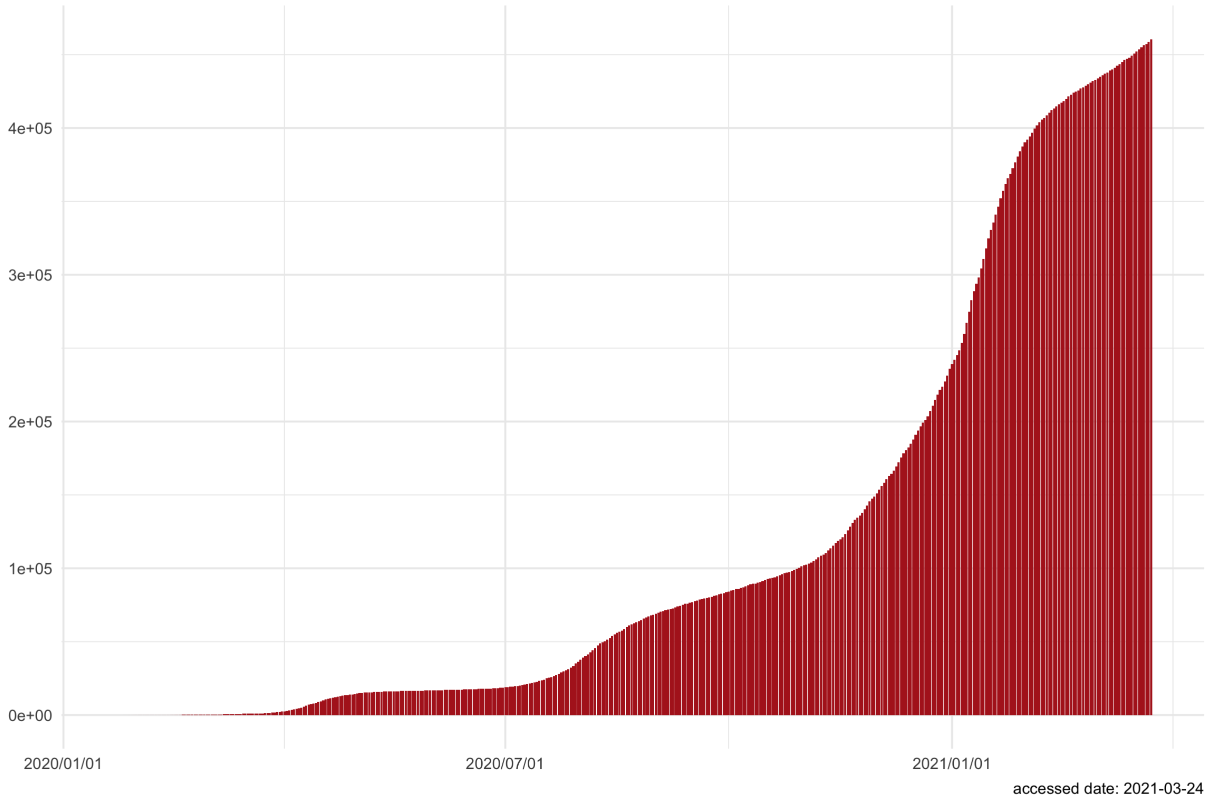

Plot the trend for for the Top 10 increase cases countries on last day

```
library("dplyr")
library("ggrepel")

x <- res$latest
y <- res$historical

country_list =  x["global"]$country[1:10]

y[country_list]  %>%
subset( date > as.Date("2020-10-01") ) %>%
group_by(country) %>%
arrange(country,date) %>%
mutate(increase = cases - lag(cases, default =  first(cases))) -> df

ggplot(df, aes(x=date, y=increase, color=country  ))+
  geom_smooth() +
  geom_label_repel(aes(label = paste(country,increase)),
    data = df[df$date == max(df$date), ], hjust = 1) +
  labs(x=NULL,y=NULL)+
  theme_bw() + theme(legend.position = 'none')
```

```
## `geom_smooth()` using method = 'loess' and formula 'y ~ x'
```

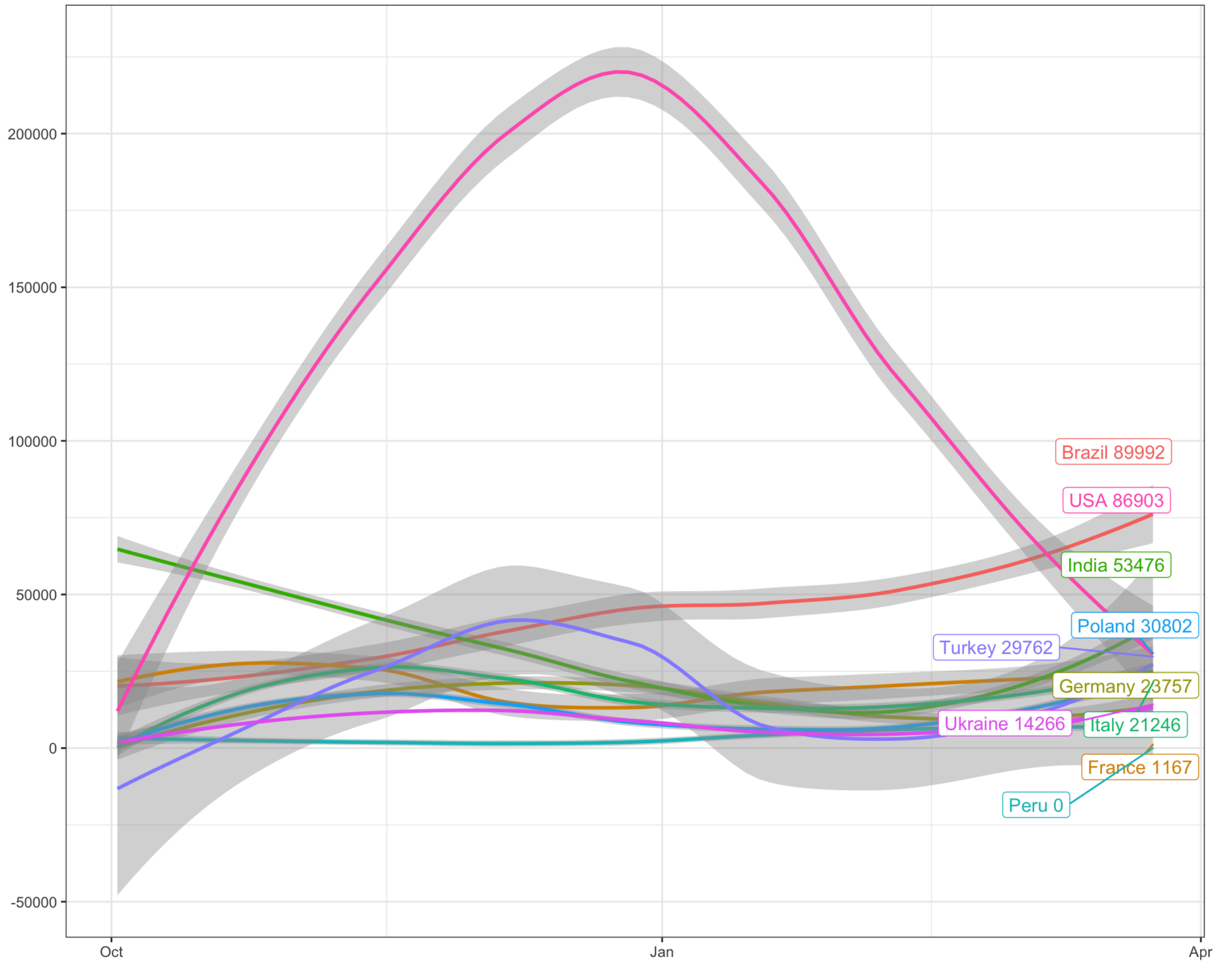

Plot the curve of cases, recovered and deaths for specify country

```
library('tidyr')
library('ggrepel')
library('ggplot2')
y <- res$historical
country = "India"

y[country] -> d
d <- gather(d, curve, count, -date, -country)

ggplot(d, aes(date, count, color = curve)) + geom_point() + geom_line() +
  labs(x=NULL,y=NULL,title=paste("Trend of cases, recovered and deaths in",
country)) +
    scale_color_manual(values=c("#f39c12", "#dd4b39", "#00a65a")) +
    theme_bw() +
  geom_label_repel(aes(label = paste(curve,count)),
                   data = d[d$date == max(d$date), ], hjust = 1) +
  theme(legend.position = "none",
        axis.text = element_text(angle = 15, hjust = 1)) +
  scale_x_date(date_labels = "%Y-%m-%d")
```

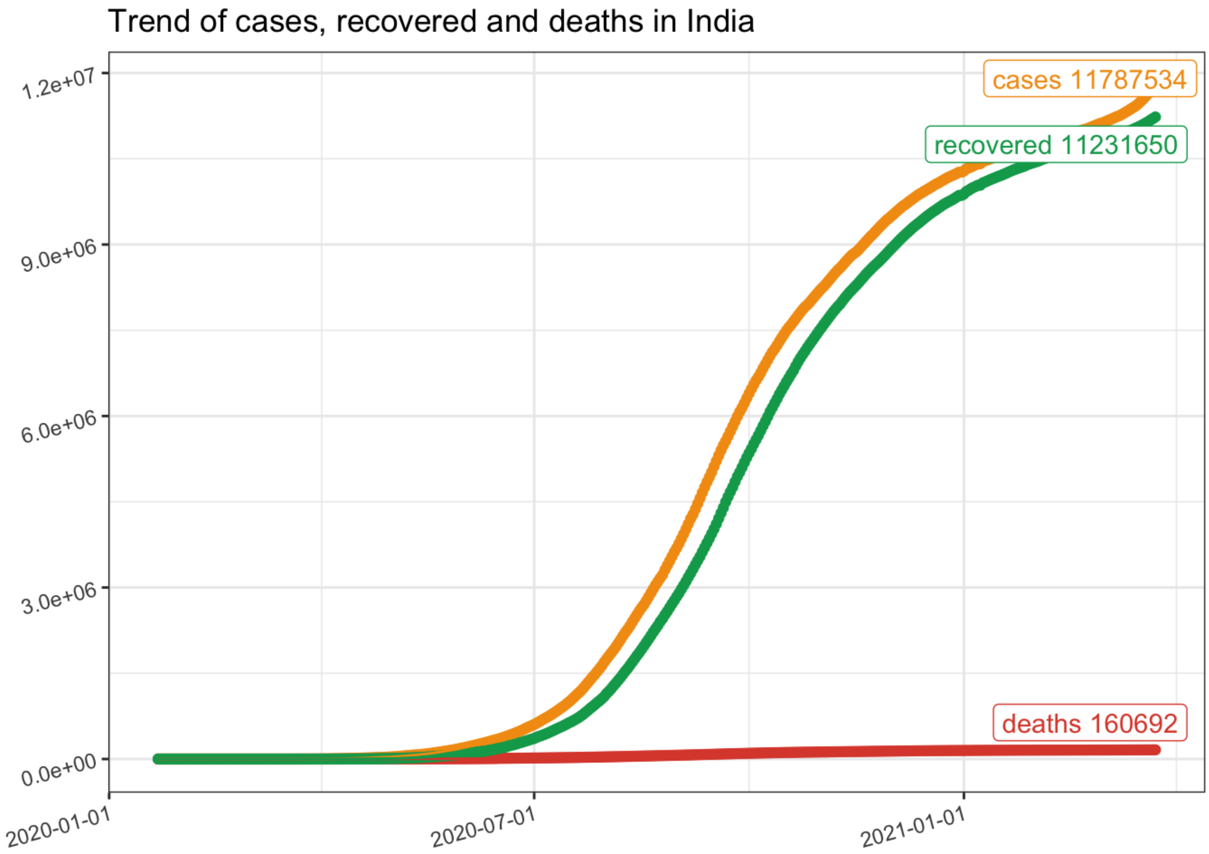

Heatmap for cases per country

Here is the example code for draw a heatmap for the historical data range in nCov2019.

```
library('tidyr')
library('ggrepel')
library('ggplot2')
y <- res$historical
d <- y["global"]

d <- d[d$cases > 0,]
length(unique(d$country))
```

```
## [1] 192
```

```
d <- subset(d,date <= as.Date("2020-3-19"))
max_time <- max(d$date)
min_time <- max_time - 7
d <-  d[d$date >= min_time,]
dd <- d[d$date == max(d$date,na.rm = TRUE),]

d$country <- factor(d$country,
  levels=unique(dd$country[order(dd$cases)]))
breaks = c(0,1000, 10000, 100000, 10000000)

ggplot(d, aes(date, country)) +
  geom_tile(aes(fill = cases), color = 'black') +
  scale_fill_viridis_c(trans = 'log', breaks = breaks,
  labels = breaks) +
  xlab(NULL) + ylab(NULL) +
  scale_x_date(date_labels = "%Y-%m-%d") + theme_minimal()
```

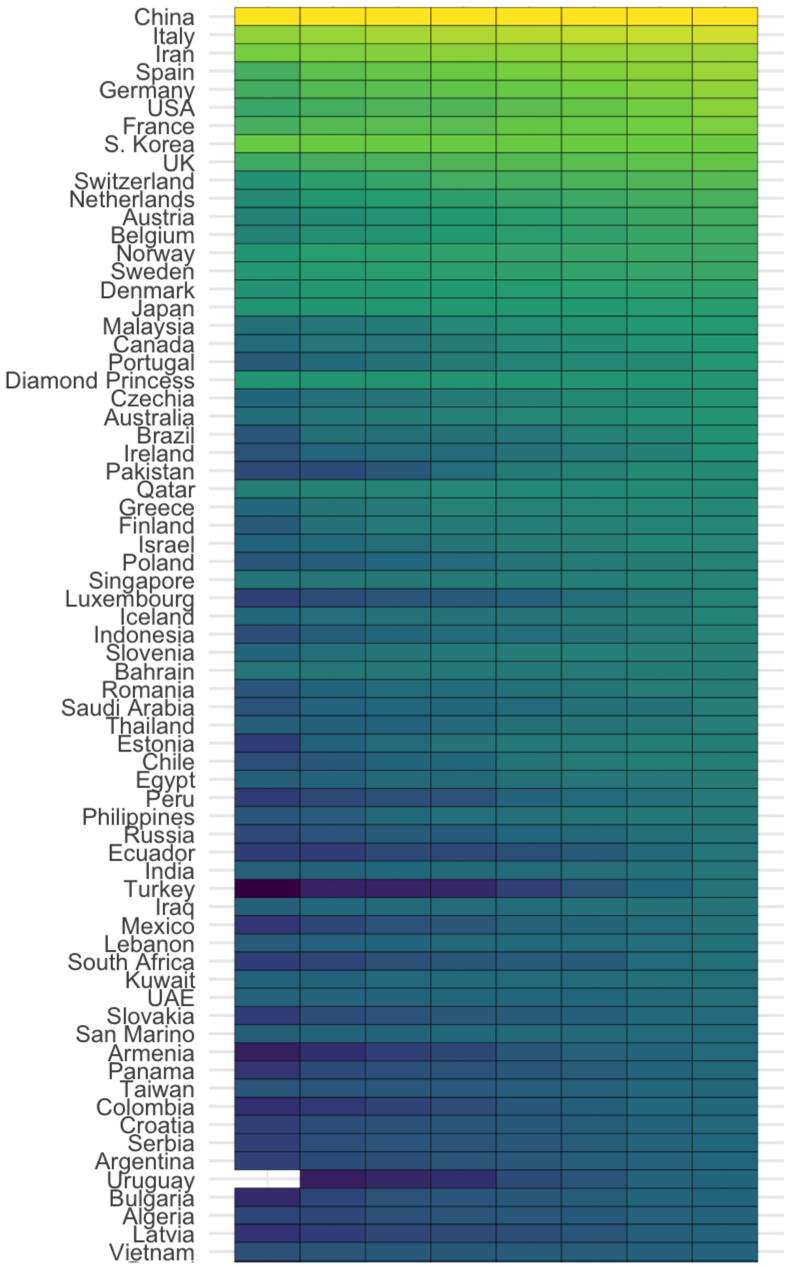

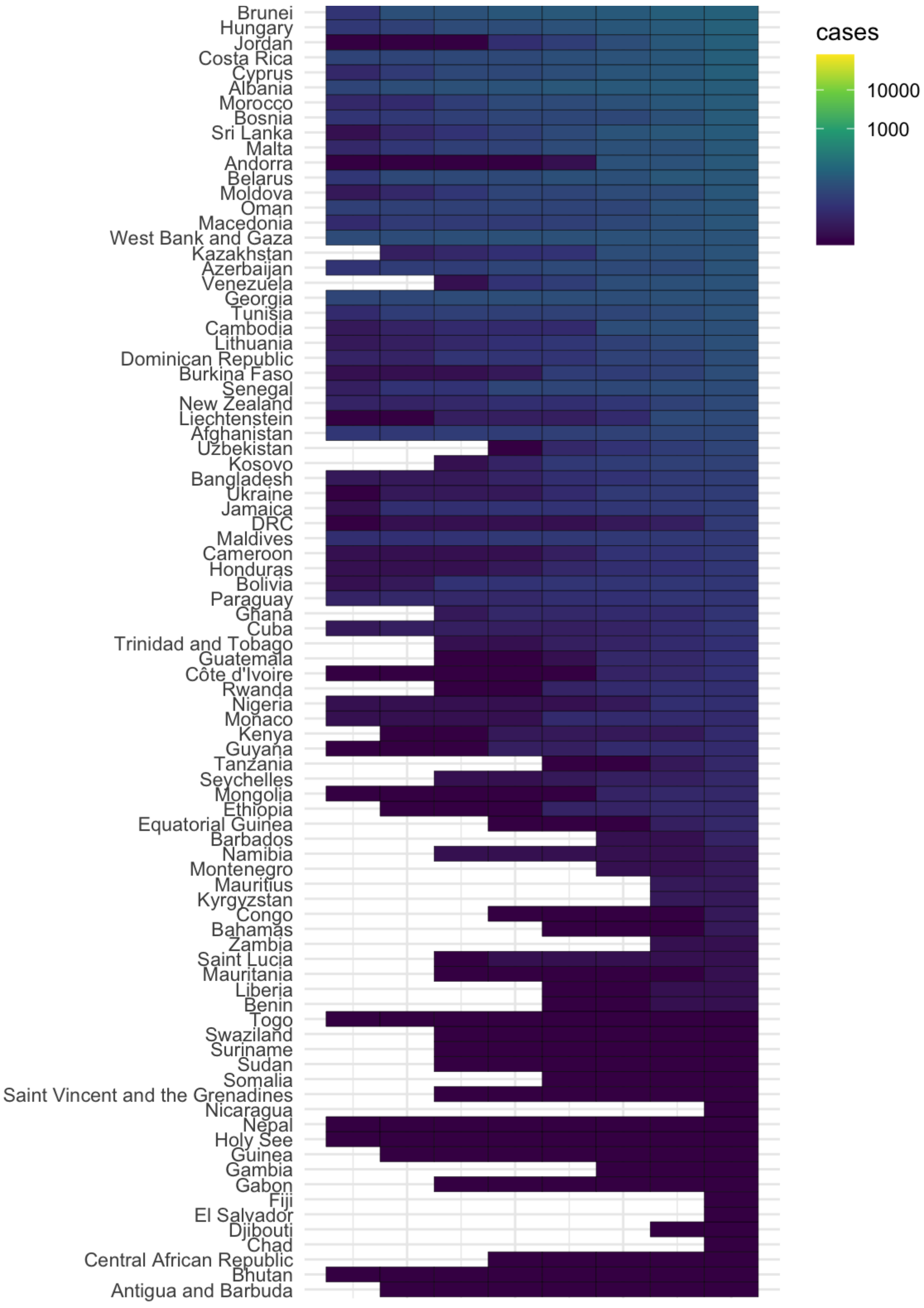

Plot the global trend in a novel way.

```
require(dplyr)

y <- res$historical
d <- y["global"]

time = as.Date("2020-03-19")
dd <- filter(d, date == time) %>%
    arrange(desc(cases))

dd = dd[1:40, ]
dd$country = factor(dd$country, levels=dd$country)

dd$angle = 1:40 * 360/40
require(ggplot2)
p <- ggplot(dd, aes(country, cases, fill=cases)) +
    geom_col(width=1, color='grey90') +
    geom_col(aes(y=I(5)), width=1, fill='grey90', alpha = .2) +
    geom_col(aes(y=I(3)), width=1, fill='grey90', alpha = .2) +
    geom_col(aes(y=I(2)), width=1, fill = "white") +
    scale_y_log10() +
    scale_fill_gradientn(colors=c("darkgreen", "green", "orange", "firebric
k","red"), trans="log") +
    geom_text(aes(label=paste(country, cases, sep="\n"),
                  y = cases *.8, angle=angle),
            data=function(d) d[d$cases > 700,],
            size=3, color = "white", fontface="bold", vjust=1)  +
     geom_text(aes(label=paste0(cases, " cases ", country),
                  y = max(cases) * 2, angle=angle+90),
            data=function(d) d[d$cases < 700,],
            size=3, vjust=0) +
    coord_polar(direction=-1) +
    theme_void() +
    theme(legend.position="none") +
    ggtitle("COVID19 global trend", time)
p
```

COVID19 global trend
2020-03-19

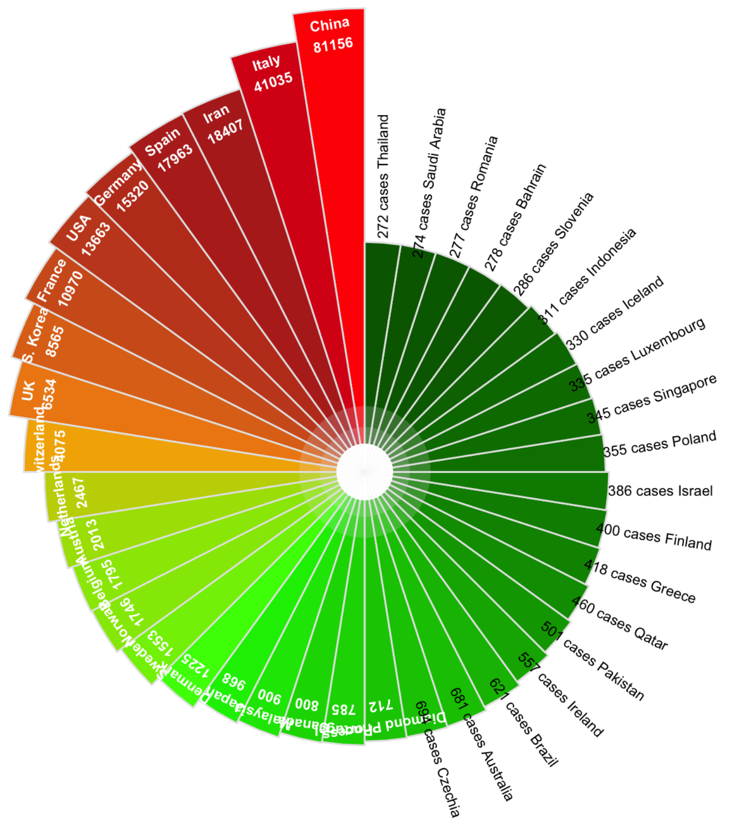

Number of days since 1 million cases per country

```
require(dplyr)
require(ggplot2)
require(shadowtext)
```

```
## Loading required package: shadowtext
```

```
y <- res$historical
d <- y["global"]



dd <- d %>%
  as_tibble %>%
  filter(cases > 1000000) %>%
  group_by(country) %>%
  mutate(days_since_1m = as.numeric(date - min(date))) %>%
  ungroup




breaks=c(1000, 10000, 20000, 50000, 500000,500000,5000000,20000000)


p <- ggplot(dd, aes(days_since_1m, cases, color = country)) +
  geom_smooth(method='lm', aes(group=1),
              data = dd,
              color='grey10', linetype='dashed') +
  geom_line(size = 0.8) +
  geom_point(pch = 21, size = 1) +
  scale_y_log10(expand = expansion(add = c(0,0.1)),
                breaks = breaks, labels = breaks) +
  scale_x_continuous(expand = expansion(add = c(0,1))) +
  theme_minimal(base_size = 14) +
  theme(
    panel.grid.minor = element_blank(),
    legend.position = "none",
    plot.margin = margin(3,15,3,3,"mm")
  ) +
  coord_cartesian(clip = "off") +
  geom_shadowtext(aes(label = paste0(" ",country)), hjust=0, vjust = 0,
                  data = . %>% group_by(country) %>% top_n(1,days_since_1
m),
                  bg.color = "white") +
  labs(x = "Number of days since 1,000,000th case", y = "",
       subtitle = "Total number of cases")
print(p)
```

```
## `geom_smooth()` using formula 'y ~ x'
```

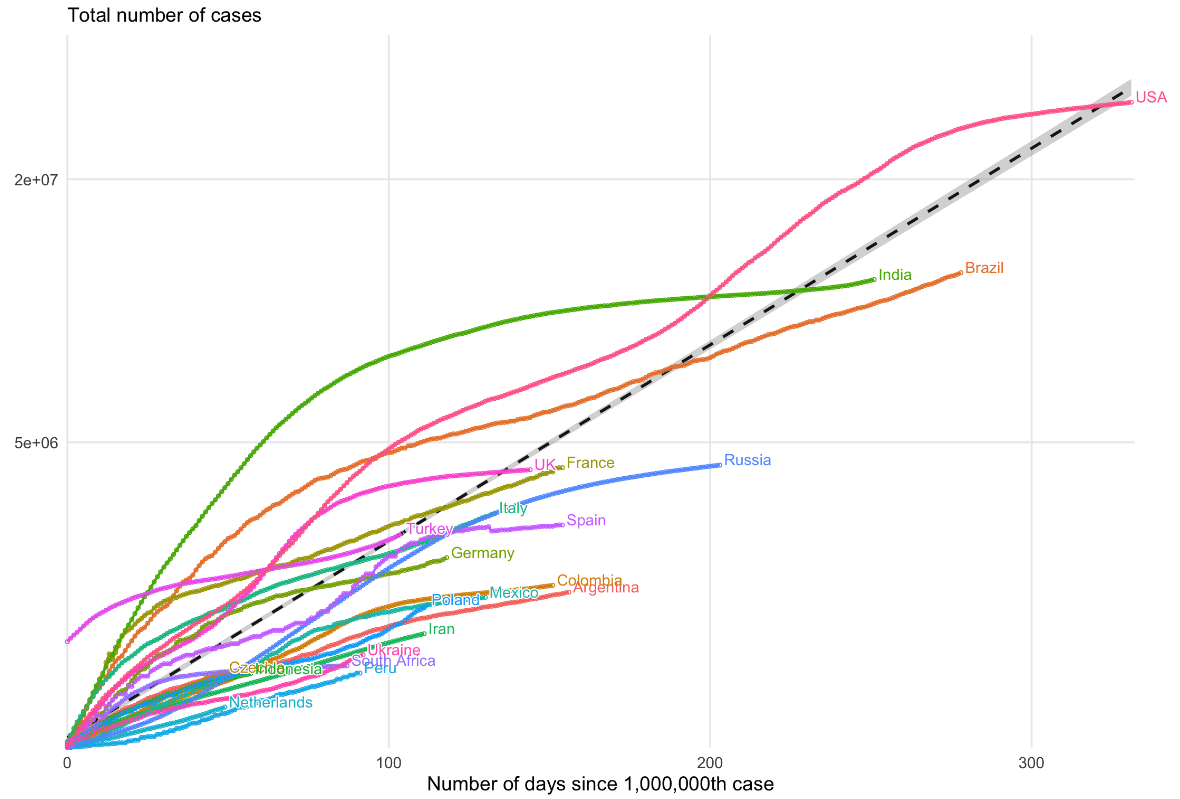

Dashboard

dashboard could launch as below:

```
dashboard()
```

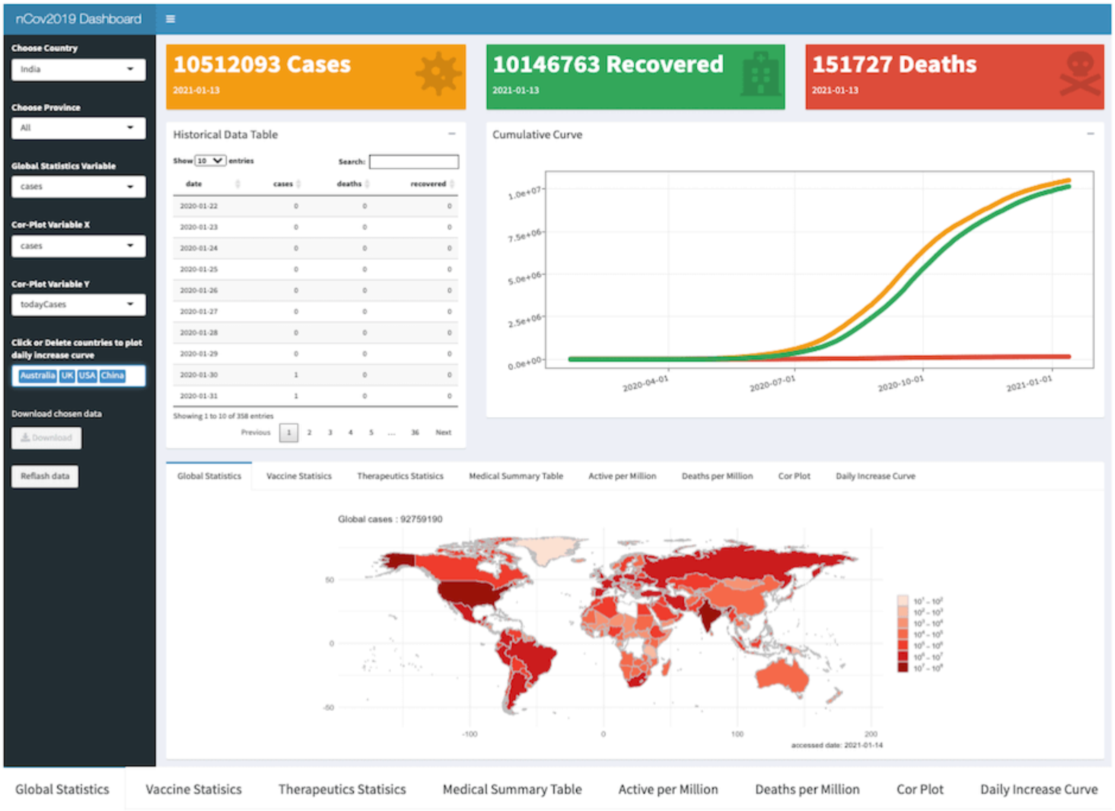

dashboard

statistic item explanation

statistic	explain
active	active number = comfirmed cases - deaths - recoveredd
activePerOneMillion	active number / million population
cases	comfirmed cases
casesPerOneMillion	comfirmed cases / million population
continent	continent
country	country
critical	Critical patients
criticalPerOneMillion	Critical patients / million population
date	date
deaths	deaths
deathsPerOneMillion	deaths patients / million population
oneCasePerPeople	oneCasePerPeople

statistic	explain
oneDeathPerPeople	oneDeathPerPeople
oneTestPerPeople	oneTestPerPeople
population	population
recovered	recovered
recoveredPerOneMillion	recoveredPerOneMillion
tests	COVID-19 test
testsPerOneMillion	COVID-19 test / million population
todayCases	comfirm cases in today
todayDeaths	comfirm cases in today
todayRecovered	comfirm cases in today
updated	the latest update time

Session Info

```
sessionInfo()
```

```
## R version 4.0.2 (2020-06-22)
## Platform: x86_64-apple-darwin19.5.0 (64-bit)
## Running under: macOS Catalina 10.15.6
##
## Matrix products: default
## BLAS/LAPACK: /usr/local/Cellar/openblas/0.3.10_1/lib/libopenblasp-r0.3.1
0.dylib
##
## locale:
## [1] en_US.UTF-8/en_US.UTF-8/en_US.UTF-8/C/en_US.UTF-8/en_US.UTF-8
##
## attached base packages:
## [1] stats     graphics  grDevices utils     datasets  methods   base
##
## other attached packages:
## [1] shadowtext_0.0.7 tidyr_1.1.2      ggrepel_0.8.2    dplyr_1.0.2
## [5] ggplot2_3.3.2    nCov2019_0.4.0
##
## loaded via a namespace (and not attached):
##  [1] Rcpp_1.0.5         RColorBrewer_1.1-2 pillar_1.4.6       compiler_
4.0.2
##  [5] plyr_1.8.6         tools_4.0.2        digest_0.6.25      viridisLit
e_0.3.0
##  [9] lattice_0.20-41    nlme_3.1-148       jsonlite_1.7.1     evaluate_
0.14
## [13] lifecycle_0.2.0    tibble_3.0.3       gtable_0.3.0       mgcv_1.8-3
1
## [17] pkgconfig_2.0.3    rlang_0.4.7        Matrix_1.2-18      yaml_2.2.1
## [21] xfun_0.20          withr_2.2.0        downloader_0.4     stringr_1.
4.0
## [25] knitr_1.30         generics_0.0.2     vctrs_0.3.4        maps_3.3.0
## [29] grid_4.0.2         tidyselect_1.1.0   glue_1.4.2         R6_2.4.1
## [33] rmarkdown_2.3      farver_2.0.3       purrr_0.3.4        reshape2_
1.4.4
## [37] magrittr_1.5       splines_4.0.2      scales_1.1.1       ellipsis_
0.3.1
## [41] htmltools_0.5.0    colorspace_1.4-1   labeling_0.3       stringi_1.
5.3
## [45] munsell_0.5.0      crayon_1.3.4
```
