# Supplementary figures and images for: nCov2019: an R package for studying the COVID-19 coronavirus pandemic
Source: PeerJ. 2021 Jun 10;9:e11421. doi: 10.7717/peerj.11421 (PMC8199916; doi:10.7717/peerj.11421)

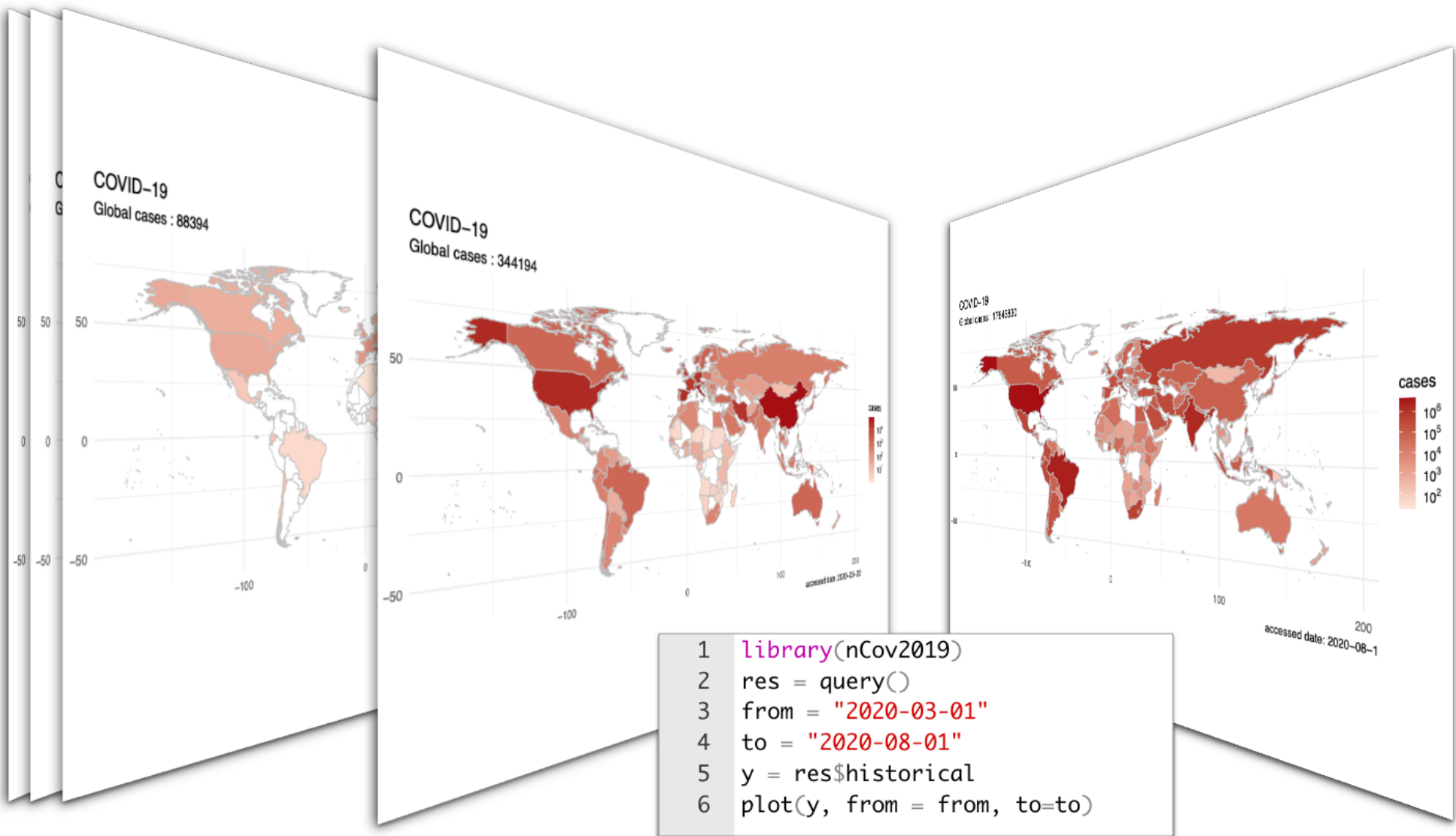

Supplement: Supplemental Information 2 — The set of maps in a specified time range can be plotted by using simple commands as shown at the center. [file peerj-09-11421-s002.pdf]

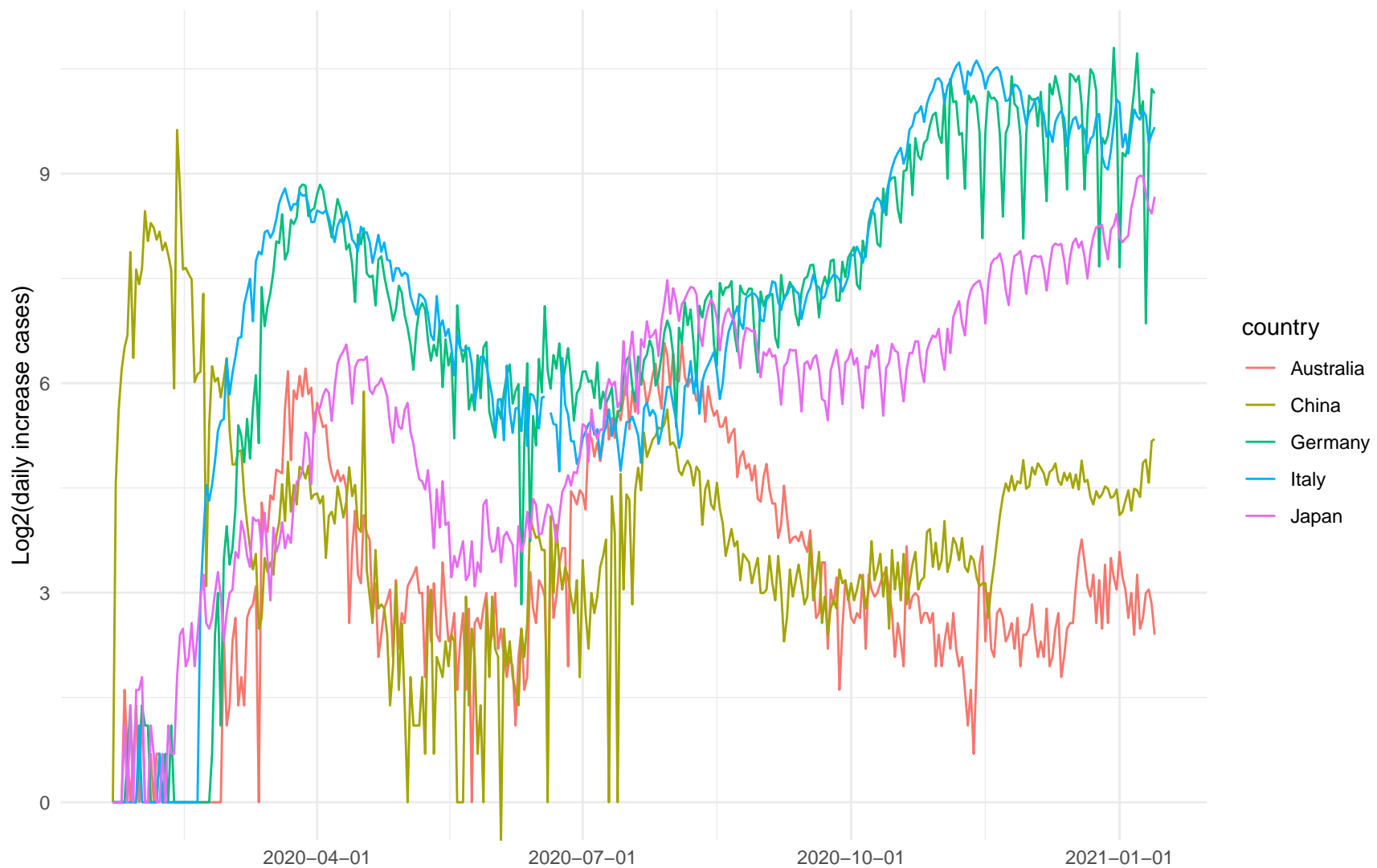

Supplement: Supplemental Information 3 — Similar charts can be explored interactively on the dashboard. [file peerj-09-11421-s003.pdf]
